# Early-phase [^18^F]PI-2620 tau-PET imaging as a surrogate marker of neuronal injury

**DOI:** 10.1007/s00259-020-04788-w

**Published:** 2020-04-21

**Authors:** Leonie Beyer, Alexander Nitschmann, Henryk Barthel, Thilo van Eimeren, Marcus Unterrainer, Julia Sauerbeck, Ken Marek, Mengmeng Song, Carla Palleis, Gesine Respondek, Jochen Hammes, Michael T. Barbe, Özgür Onur, Frank Jessen, Dorothee Saur, Matthias L. Schroeter, Jost-Julian Rumpf, Michael Rullmann, Andreas Schildan, Marianne Patt, Bernd Neumaier, Olivier Barret, Jennifer Madonia, David S. Russell, Andrew W. Stephens, Sigrun Roeber, Jochen Herms, Kai Bötzel, Johannes Levin, Joseph Classen, Günter U. Höglinger, Peter Bartenstein, Victor Villemagne, Alexander Drzezga, John Seibyl, Osama Sabri, Matthias Brendel

**Affiliations:** 1 Department of Nuclear Medicine, University Hospital of Munich LMU Munich, Marchioninstraße 15, 81377 Munich, Germany; 2 Department of Nuclear Medicine, University of Leipzig, Leipzig, Germany; 3 Cognitive Neuroscience, Institute for Neuroscience and Medicine (INM-3), Research Centre Juelich, Juelich, Germany; 4 Department of Nuclear Medicine, University Hospital Cologne, Cologne, Germany; 5 Department of Neurology, University of Cologne, Faculty of Medicine and University Hospital Cologne, Cologne, Germany; 6 German Center for Neurodegenerative Diseases (DZNE), Bonn, Germany; 7 InviCRO, LLC, Boston, MA USA; 8 Molecular Neuroimaging, inviCRO, New Haven, CT USA; 9 Department of Neurology, University Hospital of Munich, LMU Munich, Munich, Germany; 10 German Center for Neurodegenerative Diseases (DZNE), Munich, Germany; 11 Department of Psychiatry, University Hospital Cologne, Cologne, Germany; 12 Center for Memory Disorders, University Hospital Cologne, Cologne, Germany; 13 Department of Neurology, University of Leipzig Medical Center, Leipzig, Germany; 14 Clinic for Cognitive Neurology, University of Leipzig, Leipzig, Germany; 15 LIFE - Leipzig Research Center for Civilization Diseases, University of Leipzig, Leipzig, Germany; 16 Max- Planck-Institute of Human Cognitive and Brain Sciences, Leipzig, Germany; 17 Institute of Neuroscience and Medicine, Nuclear Chemistry (INM-5), Forschungszentrum Juelich GmbH, Juelich, Germany; 18 Institute of Radiochemistry and Experimental Molecular Imaging, University Clinic Cologne, Cologne, Germany; 19 Life Molecular Imaging GmbH, Berlin, Germany; 20 Center for Neuropathology and Prion Research, University Hospital of Munich, LMU Munich, Munich, Germany; 21 Department of Neurology, Technische Universität München, Munich, Germany; 22 Department of Neurology, Hannover Medical School, Hannover, Germany; 23 Munich Cluster for Systems Neurology (SyNergy), Munich, Germany; 24 Department of Molecular Imaging & Therapy, Austin Health, Heidelberg, VIC Australia; 25 The Florey Institute of Neuroscience and Mental Health, The University of Melbourne, Melbourne, VIC Australia; 26 Department of Medicine, Austin Health, The University of Melbourne, Melbourne, VIC Australia

**Keywords:** Tau, PET, [^18^F]PI-2620, Perfusion, Neuronal injury

## Abstract

**Purpose:**

Second-generation tau radiotracers for use with positron emission tomography (PET) have been developed for visualization of tau deposits in vivo. For several β-amyloid and first-generation tau-PET radiotracers, it has been shown that early-phase images can be used as a surrogate of neuronal injury. Therefore, we investigated the performance of early acquisitions of the novel tau-PET radiotracer [^18^F]PI-2620 as a potential substitute for [^18^F]fluorodeoxyglucose ([^18^F]FDG).

**Methods:**

Twenty-six subjects were referred with suspected tauopathies or overlapping parkinsonian syndromes (Alzheimer’s disease, progressive supranuclear palsy, corticobasal syndrome, multi-system atrophy, Parkinson’s disease, multi-system atrophy, Parkinson's disease, frontotemporal dementia) and received a dynamic [^18^F]PI-2620 tau-PET (0–60 min p.i.) and static [^18^F]FDG-PET (30–50 min p.i.). Regional standardized uptake value ratios of early-phase images (single frame SUVr) and the blood flow estimate (R_1_) of [^18^F]PI-2620-PET were correlated with corresponding quantification of [^18^F]FDG-PET (global mean/cerebellar normalization). Reduced tracer uptake in cortical target regions was also interpreted visually using 3-dimensional stereotactic surface projections by three more and three less experienced readers. Spearman rank correlation coefficients were calculated between early-phase [^18^F]PI-2620 tau-PET and [^18^F]FDG-PET images for all cortical regions and frequencies of disagreement between images were compared for both more and less experienced readers.

**Results:**

Highest agreement with [^18^F]FDG-PET quantification was reached for [^18^F]PI-2620-PET acquisition from 0.5 to 2.5 min p.i. for global mean (lowest *R* = 0.69) and cerebellar scaling (lowest *R* = 0.63). Correlation coefficients (summed 0.5–2.5 min SUVr & R_1_) displayed strong agreement in all cortical target regions for global mean (R_SUVr_ 0.76, *R*_R1_ = 0.77) and cerebellar normalization (R_SUVr_ 0.68, *R*_R1_ = 0.68). Visual interpretation revealed high regional correlations between early-phase tau-PET and [^18^F]FDG-PET. There were no relevant differences between more and less experienced readers.

**Conclusion:**

Early-phase imaging of [^18^F]PI-2620 can serve as a surrogate biomarker for neuronal injury. Dynamic imaging or a dual time-point protocol for tau-PET imaging could supersede additional [^18^F]FDG-PET imaging by indexing both the distribution of tau and the extent of neuronal injury.

**Electronic supplementary material:**

The online version of this article (10.1007/s00259-020-04788-w) contains supplementary material, which is available to authorized users.

## Introduction

Tauopathies consist of neurodegenerative diseases including, among others, Alzheimer’s disease (AD), frontotemporal dementia and atypical parkinsonian syndromes such as progressive supranuclear palsy (PSP) and corticobasal syndrome (CBS), with the connective characteristic of misfolded and accumulated tau protein in different parts of the brain [1, 2]. Visualization of tau deposits in vivo has become possible with various tau-targeting ligands for use with positron emission tomography (PET) [3, 4]. While first-generation tau tracers suffered from off-target binding [5, 6] and a resulting large inter- and intra-case variability [7], second-generation tau-PET tracers showed promising first results in vivo with specific binding in affected regions in patients with mild cognitive impairment and AD compared to healthy controls [8–11].

Tau-PET imaging complements an important biomarker for the characterization of neurodegenerative diseases. For AD, it has been proposed to classify the disease according to the biomarkers for amyloid, tau and neuronal injury by the A/T/N scheme [12]. In this classification scheme, neuronal injury in the pathological definition can be determined in vivo by three different diagnostic approaches. Atrophy in structural magnetic resonance tomography and total tau in cerebrospinal fluid are considered as well as hypometabolism in ^18^F-fluorodeoxyglucose ([^18^F]FDG)-PET. This is underpinned by a combined study in prion disease, indicating that metabolic imaging via [^18^F]FDG correlates with neuropathologic changes including neuronal loss [13].

For first-generation tau tracers and several tracers for amyloid-PET imaging, it has been shown that the perfusion-phase images as obtained by these tracers is comparable to glucose metabolism as assessed by [^18^F]FDG-PET and can therefore be used as surrogate biomarker of neuronal injury [14–21]. With respect to cost and radiation exposure, such “one-stop-shop” protocols have the opportunity to examine two important biological markers with one procedure.

In contrast to amyloid imaging where the subjects can only be classified as amyloid-positive or amyloid-negative, tau-PET imaging shows characteristic patterns for several different neurodegenerative entities [3]. Therefore, tau-PET imaging could probably also be used to discriminate a range of tauopathies beyond AD. Due to the lack of specificity of first-generation tau tracers, it is of great interest whether and how the perfusion-phase images as obtained by second-generation tracers can be used as a marker of neuronal injury.

Thus, we aimed to investigate the potential of the second-generation tau-PET ligand [^18^F]PI-2620 [22] as an additional (in addition to detecting tau pathology) biomarker of neuronal injury. We validated early-phase [^18^F]PI-2620 data against [^18^F]FDG-PET and focused on optimizing dynamic or coffee break acquisition protocols for tau-PET imaging with dual biomarker information.

## Materials and methods

### Study design and patient enrolment

Patients with different suspected tauopathies were referred by dementia or movement disorder experts to [^18^F]PI-2620 tau-PET imaging. We selected all subjects with an additional [^18^F]FDG-PET acquired < 12 months before/after tau-PET imaging. The cohort consisted of patients with different suspected clinical diagnoses and represented a true clinical cross-section in a tertiary centre with a focus on dementia/ movement disorders. The most likely clinical differential diagnosis was recorded before [^18^F]PI-2620 tau-PET imaging. All patients provided informed written consent to PET imaging. The study was conducted in accordance with the principles of the Declaration of Helsinki, and approval for scientific data analysis was obtained from the local ethics committee (application number 17–569).

### PET imaging

#### Radiosynthesis

Radiosynthesis of [^18^F]PI-2620 was achieved by nucleophilic substitution on a BOC-protected nitro precursor using an automated synthesis module (IBA, Synthera). The protecting group was cleaved under the radiolabelling conditions. The product was purified by semipreparative HPLC. Radiochemical purity was 99%. Non-decay corrected yields were about 35% with a molar activity of 8∙10^6^ GBq/mmol at the end of synthesis. [^18^F]FDG was purchased commercially.

#### PET acquisition and preparation

All patients were scanned at the Department of Nuclear Medicine, LMU Munich, with a Biograph 64 or a Siemens mCT PET/CT scanner (both Siemens, Erlangen, Germany). A low-dose CT scan preceded the PET acquisition and served for attenuation correction. [^18^F]PI-2620-PET was performed in a full dynamic 0–60-min setting initiated upon intravenous injection (~ 10 s) of 185 ± 10 MBq of the ligand. [^18^F]PI-2620-PET data were reconstructed in a series of 23 frames (6 × 0.5 min, 4 × 1.0 min, 4 × 2.0 min, 9 × 5.0 min). [^18^F]-FDG-PET was acquired after injection of 125 ± 10 MBq [^18^F]FDG according to the EANM protocol [23]: fasting conditions > 6 h with a blood glucose < 120 mg/dl (6.7 mm) at time of scanning, silent room with dimmed light, headphones and blindfold 20 min prior and after injection. [^18^F]FDG-PET data was reconstructed in a static 30–50-min frame. PET data were reconstructed iteratively (4 iterations, 21 subsets, 5.0-mm Gauss/5 iterations, 24 subsets, 5.0-mm Gauss) with a matrix size of 336 × 336 × 109/ 400 × 400 × 148, a voxel size of 1.018 × 1.018 × 2.027/1.018 × 1.018 × 1.500 mm^3^ and a slice thickness of 2.027/1.500 mm. Standard corrections with regard to scatter, decay and random counts were used. Both reconstruction algorithms resulted in images with equal spatial resolution (8 × 8 × 7 mm) as validated by Hofmann phantom measures.

#### Image processing

All image data were processed and analysed using PMOD (version 3.5, PMOD Technologies Ltd., Zurich, Switzerland). For spatial normalization, tracer-specific templates in the MNI space were created for [^18^F]PI-2620 (30–60 min) and [^18^F]-FDG as described previously [14]. Dynamic [^18^F]PI-2620 images were coregistered to the MNI space by applying the 30–60-min transformation (brain normalization settings: nonlinear warping, 8-mm input smoothing, equal modality, 16 iterations, frequency cut-off 3, regularization 1.0, no thresholding). All images were analysed in MNI space. The regional cerebral blood flow estimate R_1_ was computed from the dynamic [^18^F]PI-2620 images by applying the simplified reference tissue model 2 (SRTM2) as described previously [24, 25], using the cerebellum (excluding the dentate nucleus and superior layers) as a reference region. A total number of ten predefined cortical volumes (bilateral frontal, central region, parietal, temporal, occipital) of interests (VOIs) deriving from the Hammers atlas [26] were delineated in the MNI space and standardized uptake value ratios (SUVr) of all VOIs were extracted for the different images (and different time frames) used for the analysis.

In preparation for visual analyses of all images (after selection of the appropriate time frame), dynamic data frames #2-#5 (0.5–2.5 min) were summed. To account for the lower count statistics of the early-phase images, an additional 6.0-mm Gaussian filter was applied for both ([^18^F]PI-2620_0.5–2.5min_ & [^18^F]PI-2620_R1_).

### PET data evaluation

#### Correlation of single frames of [^18^F]PI-2620-PET versus [^18^F]FDG-PET

The optimal early time window for [^18^F]PI-2620 early-phase imaging in terms of maximal correlation to [^18^F]FDG-PET was determined. To this end, [^18^F]PI-2620-PET SUVrs for the ten cortical VOIs were extracted and correlated with the SUVrs of the corresponding [^18^F]FDG-PET data after normalization of uptake to global mean (GBM) or by use of a cerebellar reference region (CBL). Pearson’s correlation coefficients (R) were compared between different time frames and the selection of the optimal early-phase time window was based on the extent and significance of single frame agreement.

#### Regional comparison of optimized early-phase [^18^F]PI-2620_0.5–2.5min_ and [^18^F]PI-2620_R1_ versus [^18^F]FDG

The SUVr values of the optimized summed [^18^F]PI-2620 early-phase image (0.5–2.5 min), the [^18^F]PI-2620 R_1_ image and the [^18^F]FDG image were correlated for all ten cortical regions to investigate the regional relationship between tau-PET perfusion and glucose metabolism.

#### Visual analysis of stereotactic surface projections

For visual interpretation of early-phase [^18^F]PI-2620_0.5–2.5min_, [^18^F]PI-2620_R1_ and [^18^F]FDG-PET images, three-dimensional stereotactic surface projections (3D-SSP) [27] were generated using the software Neurostat (Department of Radiology, University of Washington, Seattle, WA, USA). Voxel-wise Z-scores were calculated in Neurostat by comparing the individual tracer uptake ([^18^F]PI-2620_0.5–2.5min_, [^18^F]PI-2620_R1_ and [^18^F]FDG) to a historical [^18^F]FDG-PET database (cognitively healthy individuals, age-matched, *N* = 18).

Three more experienced nuclear medicine physicians (H.B., T.v.E., M.B.) and three less experienced nuclear medicine interns (L.B., M.U., J.S.) visually assessed the 3D-SSP images using the Z-score maps (GBM scaling) and rated cortical regions used in the clinical routine (bilateral frontal, parietal, temporal, occipital cortex areas) from 0 to 3 (0 = no reduction, 1 = low reduction, 2 = intermediate reduction, 3 = severe reduction 0.5 steps were allowed). Furthermore, whole-brain 3D-SSP images were rated binary (0/1) and according to the severity of present neurodegeneration (0 = no/1 = mild/2 = intermediate/3 = severe neurodegeneration; 0.5 steps were allowed). A significant neuronal injury of the patient (A/T/N: N+) was defined by the majority read of binarized [^18^F]FDG-PET evaluation by the three more experienced physicians. Readers had access to the pre-PET clinical diagnosis. All 3D-SSP images ([^18^F]PI-2620_0.5–2.5min_, [^18^F]PI-2620_R1_ and [^18^F]FDG) were randomly and blindly (with regard to the type of image) presented to the readers.

#### Statistical analysis

Correlations of regional SUVr between early-phase [^18^F]PI-2620 and [^18^F]FDG images were evaluated using Pearson’s correlation coefficient (R) and R values were compared using Fisher’s Z-transformation. Quantitative variables were reported as mean ± standard deviation. Comparison of R values for different normalization approaches was also performed by Fisher’s Z-transformation. For visual analysis and the specification of the most likely PET diagnosis, the intra-reader agreement between [^18^F]PI-2620_0.5–2.5min_, [^18^F]PI-2620_R1_ and [^18^F]FDG was calculated using Spearman’s rank correlation coefficients. The disagreement between visual ratings of [^18^F]PI-2620_0.5–2.5min_ or [^18^F]PI-2620_R1_ and [^18^F]FDG was calculated and evaluated as frequency of all brain regions. A significance level of *p* < 0.05 was applied in all analyses. All statistical analyses were performed using SPSS (version 25.0, IBM, Armonk, New York, USA).

## Results

### Demographics

A total of 26 subjects (age = 66 ± 11 years, 17 female) were included in the analysis. The cohort consisted of seven subjects with a most likely diagnosis of AD, 13 subjects with movement disorders and most likely diagnosis of PSP or CBS, one case with most likely frontotemporal dementia, two cases with most likely multi-system atrophy, two cases with most likely Parkinson’s disease, and one case with cognitive impairment of unknown reason. The mean time interval between both PET investigations was 1.2 ± 1.7 months. For details of the study population see Table 1. Visual binary interpretation (majority read of more experienced readers) revealed a significant neuronal injury to [^18^F]FDG-PET in 65% of all cases.

**Table 1:** Demographics of the study population

	All	Most likely AD	Most likely 4R-tauopathy	Other
Number of subjects	26	7	13	6
Age (mean ± SD)	66.0 ± 10.7	67.4 ± 9.1	70.7 ± 6.8	54.2 ± 11.6
Gender (♂/ ♀)	♂ 9/♀ 17	♂ 4/♀ 3	♂ 4/♀ 9	♂ 1/♀ 5
Time interval between PI-2620- and FDG-PET (months, mean ± SD)	1.2 ± 1.7	1.3 ± 1.8	1.2 ± 1.6	1.0 ± 2.0
Significant neuronal injury [^18^F]FDG (%, visual)	65	71	77	33

AD, Alzheimer’s disease; 4R, 4R-tauopathies (progressive supranuclear palsy, corticobasal syndrome); Other (frontotemporal dementia, multi-system atrophy, Parkinson’s disease, unclear phenotype); SD, standard deviation. Significant neuronal injury in FDG-PET was defined by the majority read of more experienced readers

### Optimal time window for [^18^F]PI-2620-PET early-phase imaging

**Fig. 1:**
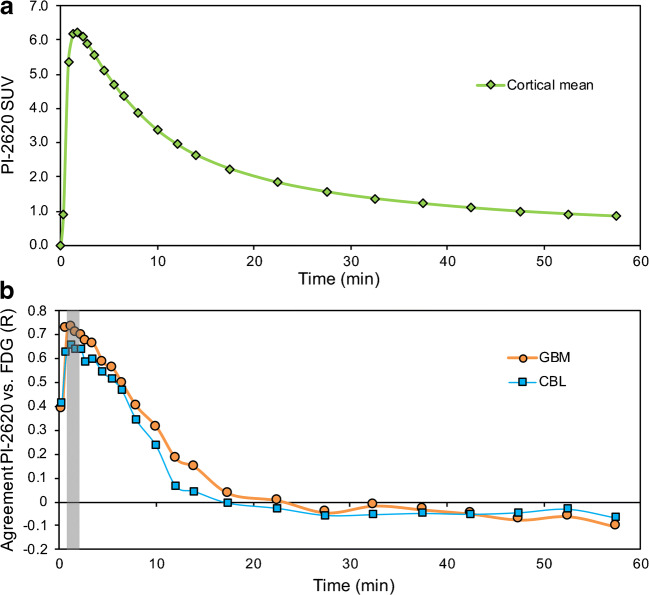
**a** Averaged (across all study subjects) time-activity curve of [^18^F]PI-2620 in a cortical composite volume of interest during the dynamic data acquisition. **b** Correlation coefficients (R) between single frames of [^18^F]PI-2620-PET and [^18^F]FDG-PET (30–50 min p.i.). GBM, global mean scaling; CBL, cerebellar reference region. The time-window used for further analyses is highlighted in grey

The VOI-based comparison of single frames of [^18^F]PI-2620-PET (6 × 0.5 min, 4 × 1.0 min, 4 × 2 min, 9 × 5.0 min) and [^18^F]FDG-PET (30–50 min p.i.) revealed the highest agreement for the frames #2–#5 lasting from 0.5–2.5 min after injection for both global mean scaling (*R*_GBM_ = 0.728) and a cerebellar reference region (*R*_CBL_ = 0.665; Fig. 1). The correlation was statistically significant for all cortical regions for frames #2–#7 for global mean scaling (0.5–4 min after injection), but frames #6–#7 showed overall lower correlation coefficients compared to frames #2–#5 (especially with a cerebellar reference region). Because of the fast wash-out and sufficient count statistics for frames #2–#5, the mean values of the 0.5–2.5 min p.i. time window (frames #2-#5) were used for further semiquantitative and visual analyses. This was as they represented the optimal trade-off between correlation with the [^18^F]FDG-PET data and count statistics. The correlation coefficients between the early [^18^F]PI-2620 and [^18^F]FDG-PET data and their degree of significance for all frames are shown in Table 2. The correlations coefficients between both normalization approaches were not significantly different (*p* = 0.301).

**Table 2:** Correlation coefficients between single frames of [^18^F]PI-2620-PET and [^18^F]FDG-PET

Time frames (min post-injection)	R	p_max_	R	p_max_
	Global mean normalization		Cerebellar normalization	
0–0.5	0.413	0.684	0.445	0.278
0.5–1.0	0.740	0.002**	0.650	0.036*
1.0–1.5	0.744	0.007**	0.679	0.019*
1.5–2.0	0.721	0.027*	0.665	0.021*
2.0–2.5	0.706	0.018*	0.667	0.041*
2.5–3.0	0.690	0.024*	0.610	0.073
3.0–4.0	0.677	0.042*	0.620	0.045*
4.0–5.0	0.599	0.071	0.550	0.225
5.0–6.0	0.573	0.225	0.519	0.086
6.0–7.0	0.506	0.520	0.452	0.140
7.0–9.0	0.405	0.493	0.309	0.294
9.0–11.0	0.322	0.692	0.184	0.954
11.0–13.0	0.190	0.984	− 0.005	0.895
13.0–15.0	0.155	0.983	− 0.018	0.995
15.0–20.0	0.040	0.968	− 0.082	0.912
20.0–25.0	0.009	0.621	− 0.110	0.875
25.0–30.0	− 0.047	0.970	− 0.149	0.873
30.0–35.0	− 0.021	0.909	− 0.137	0.961
35.0–40.0	− 0.039	0.911	− 0.136	0.850
40.0–45.0	− 0.055	0.748	− 0.144	0.702
45.0–50.0	− 0.082	0.961	− 0.139	0.937
50.0–55.0	− 0.070	0.683	− 0.126	0.983
55.0–60.0	− 0.117	0.955	− 0.162	0.804

FDG, fluorodeoxyglucose; R, correlation coefficient (Pearson, two-sided, Fisher’s Z-transformation of all regions); p_max_, maximum *p* value of all ten cortical regions. *p < 0.05, **p < 0.01

### Semiquantitative VOI-based comparison of early-phase [^18^F]PI-2620-PET and [^18^F]FDG-PET

**Fig. 2:**
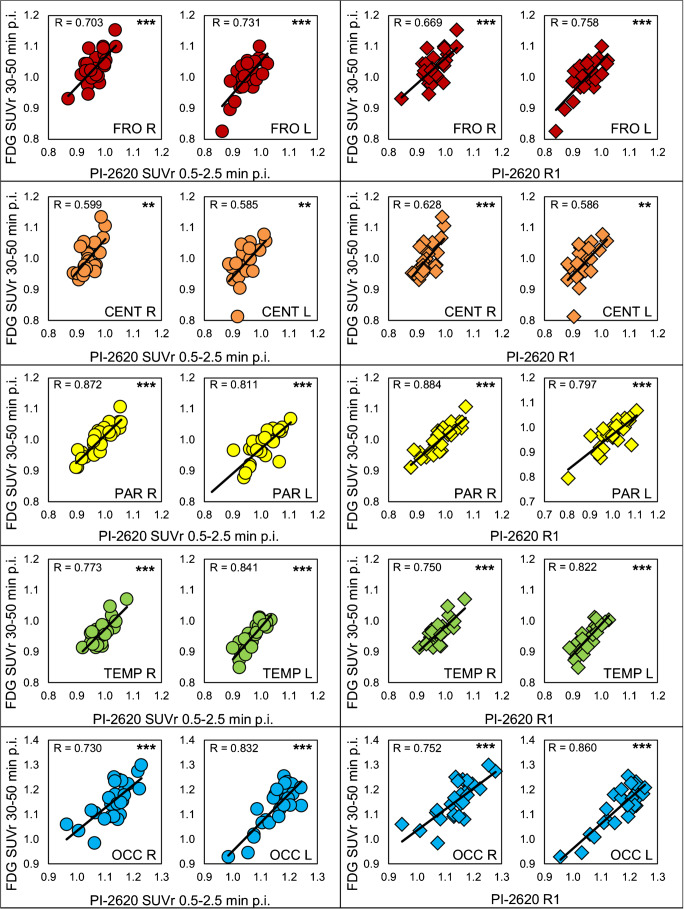
Correlation charts of early-phase [^18^F]PI-2620_0.5–2.5min_ and [^18^F]PI-2620_R1_ with [^18^F]FDG-PET SUVr (all global mean normalization). SUVr, standard-uptake-value-ratio; FRO, frontal; CENT, central; PAR, parietal; TEMP, temp; OCC, occipital

The PET parameters in all cortical brain regions showed highly significant correlations with [^18^F]FDG-PET for both early-phase [^18^F]PI-2620-PET approaches (0.5–2.5 min and R1). There was no significant difference in the correlation coefficients of [^18^F]PI-2620_0.5–2.5min_ and [^18^F]PI-2620_R1_ with [^18^F]FDG-PET (*R*_0.5–2.5min_ = 0.762, R_R1_ = 0.766, *p* = 0.487) for global mean normalization. The highest degree of correlation was found in the right parietal cortex (*R*_0.5–2.5min_ = 0.872, R_R1_ = 0.884) and the weakest correlation was observed in the left central region (*R*_0.5–2.5min_ = 0.585, *R*_R1_ = 0.586). The semiquantitative comparison of [^18^F]PI-2620_0.5–2.5min_ and [^18^F]PI-2620_R1_ with [^18^F]FDG-PET and cerebellar scaling also showed significant, but overall weaker correlations when compared to global mean normalization for all cortical regions (*R*_0.5–2.5min_ = 0.683, *R*_R1_ = 0.683, *p* = 0.472). Correlation plots for [^18^F]PI-2620_0.5–2.5min_ and [^18^F]PI-2620_R1_ with [^18^F]FDG-PET (all global mean normalization) are shown in Fig. 2. Corresponding regional values and correlation coefficients determined by comparing regional [^18^F]PI-2620_0.5–2.5min_ and [^18^F]PI-2620_R1_ with [^18^F]FDG-PET SUVr (global mean and cerebellar normalization) are shown in Supplementary Table 1.

### Visual 3D-SSP comparison of early-phase [^18^F]PI-2620-PET and [^18^F]FDG-PET

**Fig. 3:**
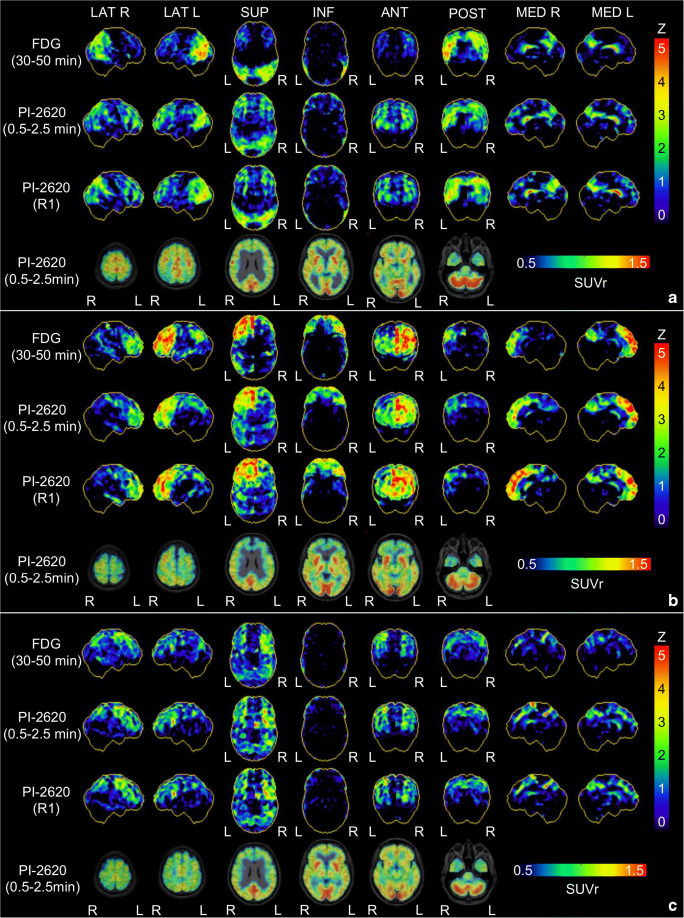
Representative 3D-SSP images (Z-score maps) of [^18^F]FDG-PET, early-phase [^18^F]PI-2620_0.5–2.5min_ and [^18^F]PI-2620_R1_ for three different most likely diagnoses of neurodegenerative disorders: **a** Alzheimer’s disease, **b** frontotemporal dementia, **c** progressive supranuclear palsy (Richardson syndrome). Surface projections from R, right; L, left; LAT, lateral; SUP, superior; INF, inferior; ANT, anterior; POST, posterior; MED, medial

After identification of the optimal time window, visual assessment was performed by evaluating 3D-SSP images of early-phase ([^18^F]PI-2620_0.5–2.5min_ and [^18^F]PI-2620_R1_) and [^18^F]FDG-PET (global mean normalization). Representative images of early-phase [^18^F]PI-2620_0.5–2.5min_, [^18^F]PI-2620_R1_ and [^18^F]FDG-PET 3D-SSP and exemplary summed 0.5–2.5-min section images for three different most likely diagnoses of neurodegenerative disorders are shown in Fig. 3. In all cases, the regional pattern of hypoperfusion in [^18^F]PI-2620_0.5–2.5min_ and [^18^F]PI-2620_R1_ 3D-SSP resembled the hypometabolism pattern in [^18^F]FDG-PET. Furthermore, exemplary distribution volume ratio images of those cases with the specific tau-PET binding pattern are shown in Supplementary Fig. 1.

Visual interpretation revealed moderate to high regional correlations for the majority of regions between early-phase tau-PET (both PI-2620_0.5–2.5min_ and PI-2620_R1_) and [^18^F]FDG-PET (see Table 3).

**Table 3:** Spearman’s rank correlation coefficients (*ρ*) between visual assessments of [^18^F]FDG-PET and early-phase [^18^F]PI-2620-PET; SUM, summed image 0.5–2.5 min after injection of [^18^F]PI-2620; LER, less experienced reader; MER, more experienced reader; R, right; L, left; ND, neurodegeneration

	LER1	LER2	LER3	MER1	MER2	MER3
FDG versus SUM	*ρ*	*ρ*	*ρ*	*ρ*	*ρ*	*ρ*
Likelihood of ND	0.661**	0.159	0.335	0.545**	0.404*	0.405*
Frontal R	0.666**	0.579**	0.407*	0.491*	0.403*	0.306
Frontal L	0.738**	0.576**	0.482*	0.550**	0.568**	0.525**
Parietal R	0.876**	0.641**	0.323	0.612**	0.398*	0.803**
Parietal L	0.717**	0.725**	0.478*	0.531**	0.359	0.659**
Temporal R	0.454*	0.517**	0.470*	0.677**	0.294	0.239
Temporal L	0.634**	0.399*	0.658**	0.566**	0.565**	0.321
Occipital R	− 0.097	0.385	0.587**	0.676**	0.074	0.692**
Occipital L	0.280	0.476*	0.566**	0.852**	0.554**	0.557**
FDG versus R1	*ρ*	*ρ*	*ρ*	*ρ*	*ρ*	*ρ*
Likelihood of ND	0.541**	0.267	0.526**	0.634**	0.598**	0.593**
Frontal R	0.366	0.447*	0.258	0.432*	0.255	0.326
Frontal L	0.732**	0.576**	0.600**	0.598**	0.558**	0.401*
Parietal R	0.756**	0.726**	0.614**	0.674**	0.508**	0.742**
Parietal L	0.617**	0.714**	0.724**	0.554**	0.654**	0.560**
Temporal R	0.577**	0.493	0.371	0.367	0.337	0.588**
Temporal L	0.670**	0.454*	0.706**	0.557**	0.747**	0.537**
Occipital R	− 0.097	0.180	− 0.002	0.820**	0.286	0.731**
Occipital L	0.345	0.509**	0.598**	0.801**	0.684**	0.728**

*p < 0.05, **p < 0.01

**Fig. 4:**
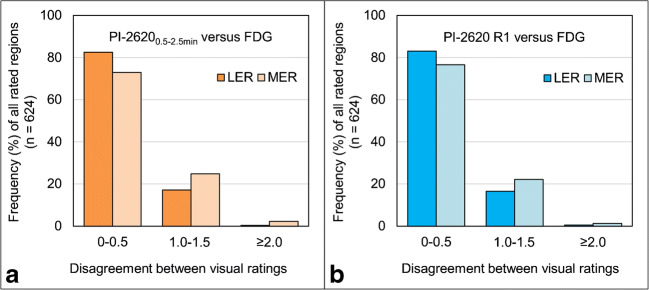
Disagreement between visual rating of early-phase **a** [^18^F]PI-2620_0.5–2.5min_ and **b** [^18^F]PI-2620_R1_ 3D-SSP images in contrast to visual rating of [^18^F]FDG-PET 3D-SSP images for all evaluated brain regions of all cases (*n* = 624). Less experienced (LER) and more experienced (MER) readers are displayed separately

The frequency of no or only minor disagreement was far higher (0–0.5; 73–83%) when compared to the frequency of moderate (1.0–1.5; 17–25%) or high disagreement (≥ 2.0; 1–2%) regardless of using PI-2620_0.5–2.5min_ or PI-2620_R1_ for early-phase assessment (Fig. 4). Frequencies of disagreement were similar for more and less experienced readers (compare Fig. 4a, b).

## Discussion

Tau-PET imaging is of great interest as tauopathies make up the majority of neurodegenerative diseases [28]. However, not only information on tau-positivity but also on the presence of neuronal injury is considered in current diagnosis or research criteria of AD [12, 29] and non-AD tauopathies [30]. In accordance with previous amyloid-PET studies [14–18, 21], and considering the known limitations of first-generation tau radiotracers [19, 31], we aimed to evaluate the potential of the early-phase of the second-generation tau-PET radiotracer [^18^F]PI-2620 [11] as a potential surrogate biomarker of neuronal injury. Our results demonstrate a strong quantitative and visual agreement between reduced perfusion in tau-PET and reduced glucose metabolism in [^18^F]FDG-PET imaging. Therefore, early-phase tau-PET images may potentially eliminate the need for an additional [^18^F]FDG-PET. The ability to obtain two different biomarkers reduces costs, time and radiation exposure, and enables improved differential diagnosis through a one-stop-shop procedure, which is especially important for disabled patients.

The semiquantitative comparison of [^18^F]PI-2620-PET single frames with individual [^18^F]FDG-PET images revealed the highest agreement at 0.5–2.5 min after injection and decreased steadily until it was no longer significant for all cortical regions later than 4 min after injection. Previous studies evaluating the agreement between early-phase amyloid-PET acquisitions and [^18^F]FDG-PET suggested time frames up to 10 min after injection, often sparing the first minute after injection [14, 15, 17]. Compared to these studies, we can conclude that the acquisition of early-phase [^18^F]PI-2620 must include a very early and relatively short time frame to achieve a high agreement between tau-PET and [^18^F]FDG-PET, likely related to the fast washout of [^18^F]PI-2620 (Fig. 1a). Although the frames between 0.5 and 4.0 min p.i. indicated a significant correlation with [^18^F]FDG-PET, we recommend the use of 0.5 to 2.5 min p.i. for early-phase [^18^F]PI-2620 acquisition as this time window was more robust across different scaling methods. For implementation in study or clinical routine workflows, dynamic or coffee break protocols (included break between early- and late-image acquisitions with the possibility for the patient to rest) could be considered. In this regard, dynamic protocols will have the advantage of being able to perform non-invasive kinetic modelling for the tau-PET read-out, which will be important to account for blood flow changes in longitudinal studies [32]. We also propose to exclude the first half minute of acquisition, which showed more variability, likely related to variance in blood flow (i.e., reduced cardiac ejection fraction), site of injection, and variance of manual injection. Therefore, we additionally calculated the regional cerebral blood flow estimate R_1_ of [^18^F]PI-2620 to take such individual differences into account and found similar results by this approach. The VOI-based semiquantitative regional correlation of both the summed early-phase (0.5–2.5 min) and R_1_ of [^18^F]PI-2620 showed a highly significant correlation in all assessed cortical regions and no significant advantage for one of the methods. For clinical implementation, the reconstruction of a summed image seems to be more suitable as it can be easily added to conventional reconstruction algorithms of common PET systems.

All semiquantitative analyses were performed with global mean normalization and whole cerebellum scaling. In accordance with our previous investigation on early-phase amyloid-PET [14], the cerebellar normalization approach showed lower correlations for all investigated cortical regions. Since cerebellar perfusion tends to be highly variable, e.g., as a consequence of significant gender differences [33], a global mean normalization is likely superior to cerebellar scaling for interpretation of early-phase tau-PET images at the level of individual patients. Nevertheless, in subjects with an overall high load of neuronal injury or other reasons of a reduced whole brain perfusion (e.g., after ischemic stroke), the cerebellar scaling proved to be a viable alternative approach for normalization.

Based on the semiquantitative results, the visual analysis of summed [^18^F]PI-2620 perfusion-like images was also performed with 3DSSP images generated by global mean normalization. Both tau-PET early-phase images (summed 0.5–2.5 min and R_1_) showed an overall high visual agreement for the detection of neuronal injury with the corresponding metabolic imaging. Less and more experienced nuclear medicine physicians had no or only minor disagreement between early-phase [^18^F]PI-2620-PET and metabolic images in the majority of cases, indicating broad clinical applicability. Different patient preparation prior to injection of [^18^F]FDG (eye patch and noise cancelling according to the EANM protocol [23]) and [^18^F]PI-2620 (room noise, no eye patch) needs to be considered as a limitation.

In our population of subjects referred with suspected tauopathies, half of all patients showed a significant neuronal injury pattern. This highlights the potential of this dual-phase protocol for evaluating two different biomarkers with one examination. While the detailed additive value of early-phase tau-PET imaging ultimately needs to be evaluated in larger samples, we already obtained promising results in individual cases. The patient with a final clinical diagnosis of frontotemporal dementia shown in Fig. 3 was initially referred with suspected AD, and the combination of tau-negativity together with a frontal hypoperfusion pattern was in line with the clinical presentation after 1-year follow-up. For the evaluation of perfusion-like images as a surrogate of neuronal injury, the comparison with metabolic imaging has the restriction that even a metabolic correlate might not unequivocally represent the actually existing neuronal injury. Temporary neuronal dysfunctions, for example, in the context of a diaschisis or neuroinflammation, cannot be differentiated from the presence of neuronal damage.

Both reduced metabolism in [^18^F]FDG-PET and reduced perfusion in perfusion-phase images of different radiotracers are influenced and partially caused by partial volume effects and might not purely reflect the underlying neuronal injury. Nevertheless, a close correlation with atrophy in magnetic resonance imaging [34] or neuropathology findings [13] was demonstrated for metabolic imaging via [^18^F]FDG. Therefore, most classification schemes, such as ATN [12], list [^18^F]FDG-PET as a surrogate of neuronal injury together with atrophy in structural magnetic resonance imaging and total tau in cerebrospinal fluid. Our findings clearly demonstrate that dual-phase [^18^F]PI-2620-PET cannot only provide information on “T” [22] but has also the potential to facilitate assessment of “N”.

## Conclusions

The present study demonstrates that early-phase imaging of second-generation [^18^F]PI-2620 tau-PET can serve as a surrogate biomarker for neuronal injury as it shows excellent semiquantitative and visual agreement with metabolic imaging using [^18^F]FDG-PET. Dynamic imaging or a dual time-point protocol for tau-PET could replace additional [^18^F]FDG-PET imaging by indexing two biomarkers in neurodegenerative disease, the distribution of tau and the amount and regional pattern of neuronal injury. The short time required for recording perfusion-like images is a great advantage in terms of patient comfort, examination time, radiation safety and cost-effectiveness.

## Electronic supplementary material

ESM 1 (DOCX 20 kb)

ESM 2 (PNG 813 kb)

High resolution image (TIFF 7005 kb)
