# Supplementary material for: Early-phase [18F]PI-2620 tau-PET imaging as a surrogate marker of neuronal injury
Source: Eur J Nucl Med Mol Imaging. 2020 Apr 21;47(12):2911–22. doi: 10.1007/s00259-020-04788-w (PMC7567714; doi:10.1007/s00259-020-04788-w)
Supplement: Supplementary file 1 — (DOCX 20 kb) [file 259_2020_4788_MOESM1_ESM.docx]

Supplement:

| Supplemental Table 1 Regional values and correlation coefficients of early-phase [^18^F]PI-2620_0.5-2.5min_ and [^18^F]PI-2620_R1_ with [^18^F]FDG-PET (global mean and cerebellar normalisation). R, right; L, left; SUVr, standard-uptake-value-ratio; SD, standard deviation. R, correlation coefficient (Pearson, two-sided). ***p < 0.001, **p < 0.01 | | | | | |
| --- | --- | --- | --- | --- | --- |
| Global mean  Region | [^18^F]FDG_30-50min_  (SUVr ± SD) | [^18^F]PI-2620_0.5-2.5min_ (SUVr ± SD) | R | [^18^F]PI-2620_R1_  (± SD) | R |
| Frontal R | 1.033 ± 0.051 | 0.966 ± 0.037 | 0.703*** | 0.965 ± 0.042 | 0.669*** |
| Frontal L | 1.002 ± 0.057 | 0.952 ± 0.039 | 0.731*** | 0.950 ± 0.044 | 0.758*** |
| Central R | 1.001 ± 0.051 | 0.943 ± 0.029 | 0.599** | 0.938 ± 0.030 | 0.628*** |
| Central L | 0.982 ± 0.055 | 0.943 ± 0.032 | 0.585** | 0.937 ± 0.034 | 0.586** |
| Parietal R | 0.998 ± 0.049 | 0.981 ± 0.049 | 0.872*** | 0.979 ± 0.056 | 0.884*** |
| Parietal L | 0.973 ± 0.060 | 1.002 ± 0.060 | 0.811*** | 1.003 ± 0.066 | 0.797*** |
| Occipital R | 1.154 ± 0.078 | 1.131 ± 0.061 | 0.730*** | 1.140 ± 0.069 | 0.752*** |
| Occipital L | 1.131 ± 0.085 | 1.155 ± 0.061 | 0.832*** | 1.168 ± 0.073 | 0.860*** |
| Temporal R | 0.962 ± 0.040 | 0.987 ± 0.034 | 0.773*** | 0.977 ± 0.035 | 0.750*** |
| Temporal L | 0.949 ± 0.044 | 0.970 ± 0.035 | 0.841*** | 0.959 ± 0.036 | 0.822*** |
| Cerebellar  Region | FDG_30-50min_  (SUVr ± SD) | PI-2620_0.5-2.5min_ (SUVr ± SD) | R | PI-2620_R1_  (± SD) | R |
| Frontal R | 1.033 ± 0.051 | 0.808 ± 0.054 | 0.559** | 0.791 ± 0.056 | 0.528** |
| Frontal L | 1.002 ± 0.057 | 0.796 ± 0.047 | 0.586** | 0.778 ± 0.050 | 0.593** |
| Central R | 1.001 ± 0.051 | 0.788 ± 0.052 | 0.557** | 0.769 ± 0.056 | 0.502** |
| Central L | 0.982 ± 0.055 | 0.788 ± 0.051 | 0.570** | 0.769 ± 0.055 | 0.558** |
| Parietal R | 0.998 ± 0.049 | 0.821 ± 0.077 | 0.714*** | 0.805 ± 0.084 | 0.725*** |
| Parietal L | 0.973 ± 0.060 | 0.838 ± 0.082 | 0.760*** | 0.824 ± 0.090 | 0.762*** |
| Occipital R | 1.154 ± 0.078 | 0.947 ± 0.091 | 0.722*** | 0.937 ± 0.099 | 0.723*** |
| Occipital L | 1.131 ± 0.085 | 0.966 ± 0.086 | 0.830*** | 0.959 ± 0.097 | 0.845*** |
| Temporal R | 0.962 ± 0.040 | 0.826 ± 0.061 | 0.641*** | 0.802 ± 0.063 | 0.647*** |
| Temporal L | 0.949 ± 0.044 | 0.812 ± 0.065 | 0.767*** | 0.788 ± 0.069 | 0.785*** |

**Supplemental Fig. 1** Representative late-phase [^18^F]PI-2620 images for the three different most likely diagnoses of neurodegenerative disorders. **a** Alzheimer’s disease, **b** frontotemporal dementia, **c** progressive supranuclear palsy (Richardson syndrome). R, right; L, left; DVR = distribution volume ratios; MRTM2 = Multilinear Reference Tissue Model 2.
