# Supplementary figures and images for: Early-phase [18F]PI-2620 tau-PET imaging as a surrogate marker of neuronal injury
Source: Eur J Nucl Med Mol Imaging. 2020 Apr 21;47(12):2911–22. doi: 10.1007/s00259-020-04788-w (PMC7567714; doi:10.1007/s00259-020-04788-w)

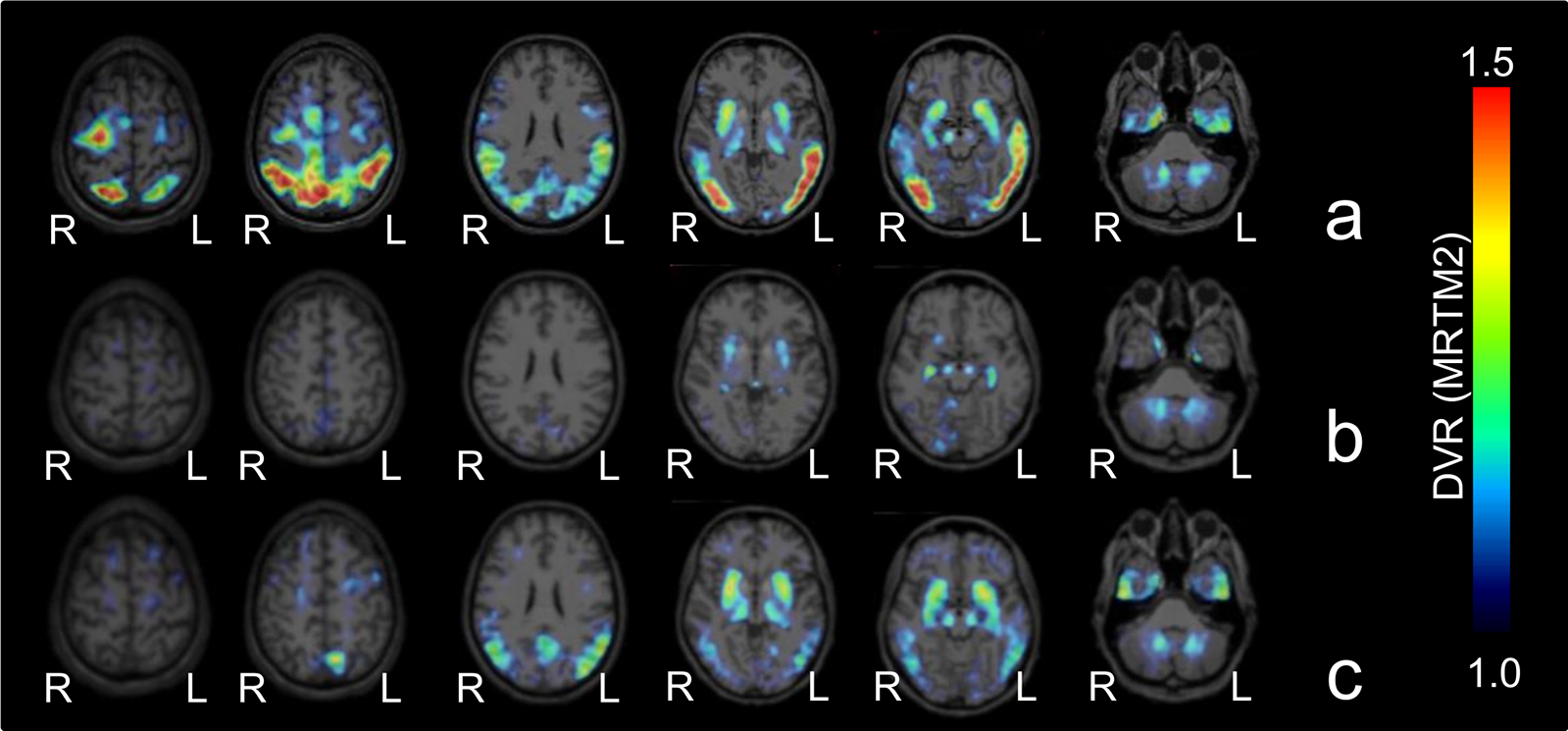

Supplement: Supplementary file 2 — (PNG 813 kb) [file 259_2020_4788_Fig5_ESM.png]

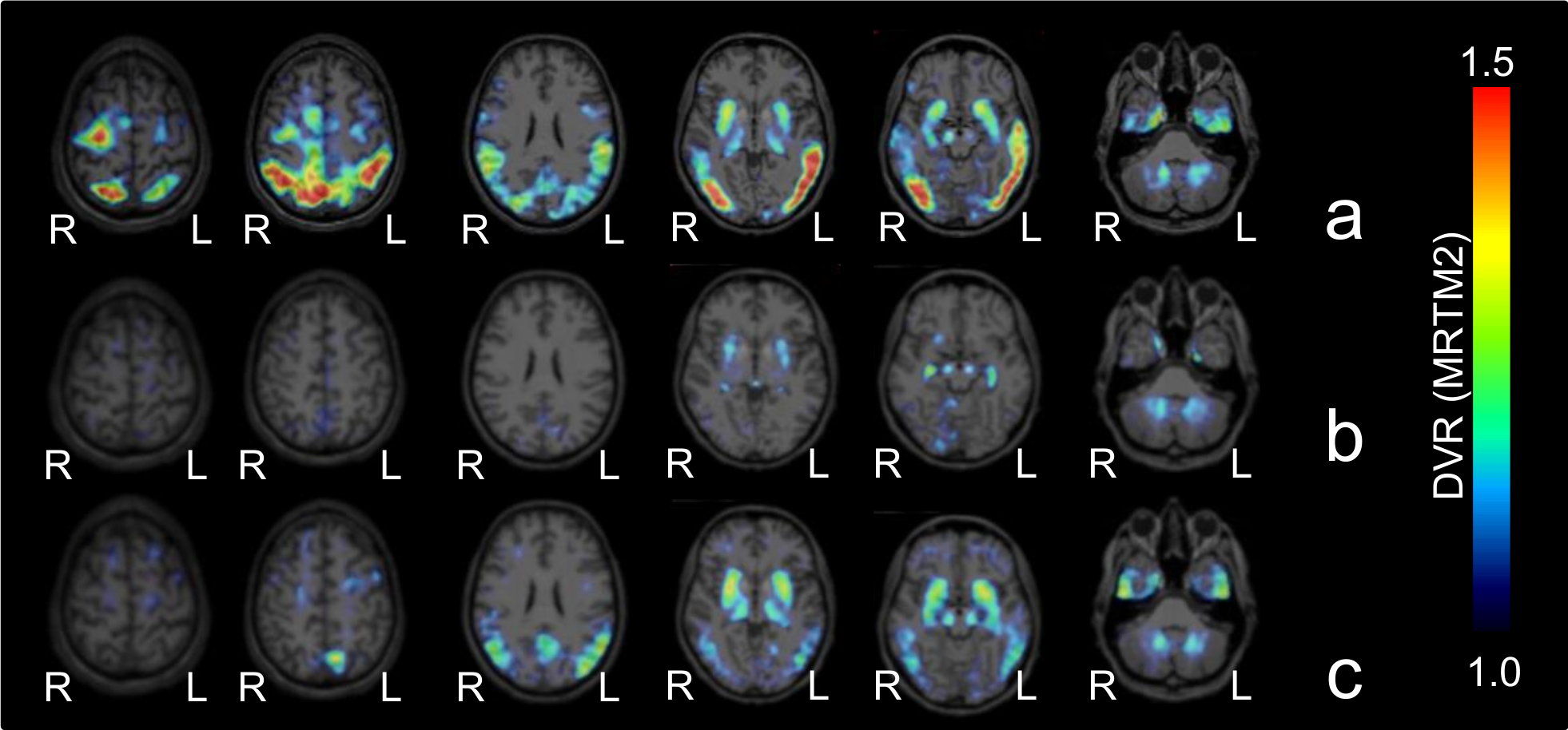

Supplement: Supplementary file 3 — High resolution image (TIFF 7005 kb) [file 259_2020_4788_MOESM2_ESM.tiff]
